# Combined Effects of PVDF/PEO-EC GEL Polymer Electrolytes for High-Performance Hybrid Electrochemical Supercapacitors

**DOI:** 10.3390/polym18040485

**Published:** 2026-02-14

**Authors:** Ramkumar Gurusamy, Tae Hwan Oh, Arunpandian Muthuraj, Aravindha Raja Selvaraj

**Affiliations:** 1 Department of Renewable Energy Science, Manonmaniam Sundaranar University, Tirunelveli 627012, Tamil Nadu, India; ramdevan92@gmail.com; 2 School of Chemical Engineering, Yeungnam University, Gyeongsan 38541, Republic of Korea; taehwanoh@ynu.ac.kr; 3 School of Chemical, Biological and Battery Engineering, Gachon University, Seongnam-si 13120, Republic of Korea

**Keywords:** ionic conductivity, micro-porous solid-state electrolyte, ethylene carbonate, hybrid supercapacitors

## Abstract

This article delineates the electrical characteristics and usefulness of a plasticized polymer electrolyte (PPE) manufactured from PVDF/PEO blends, using varying weight percentages of the plasticizer ethylene carbonate (EC) in conjunction with a liquid electrolyte. Micro-porous solid-state polymer electrolyte membranes were fabricated using the non-solvent-induced phase separation (NIPS) method. The polymer composite membranes modified by the incorporation of a plasticizer (40 weight percent of EC) exhibited enhanced porosity and absorbed a significant quantity of liquid electrolyte (313.3%). A N_2_ adsorption isotherm study indicates an increase in pore volume and pore size resulting from the incorporation of EC in PPE. This resulted in a satisfactory level of ionic conductivity (2.08 mS/cm) at 25 °C, attributable to the inclusion of 40 wt.% EC-based PPE, which has a high dielectric constant and a rapid relaxation time. The AC/40 wt.% EC-based PPE/LTO hybrid supercapacitor exhibits a superior specific capacitance, reduced internal resistance, and enhanced retention values after 10,000 cycles in comparison to the AC/10 wt.% EC-based PPE/LTO hybrid supercapacitor.

## 1. Introduction

The supercapacitor (SC) or electrochemical capacitor is a significant energy storage technology in the current energy industry, owing to its superior power density relative to rechargeable batteries. Supercapacitors (SC) are categorized by their charge storage mechanisms into electric double-layer capacitors (EDLCs), which utilize physical ion adsorption; pseudocapacitors (PCs), which depend on fast surface redox reactions; and hybrid supercapacitors (HSC), which combine both for enhanced energy and power density [1]. Currently, there is a promising trend toward utilizing renewable biomass to construct high-performance electrodes, as these precursors offer natural hierarchical porosity and heteroatom self-doping [2]. This sustainable approach addresses the limitations of conventional carbon materials by providing cost-effective, eco-friendly scaffolds with superior specific surface areas [3]. Consequently, the development of biomass-derived porous carbons has become a pivotal strategy in engineering the next generation of efficient and green energy storage devices. Hybrid supercapacitors (HSC) have a secondary battery electrode, an electric double-layer capacitor electrode, and a lithium-based electrolyte. Consequently, research on hybrid supercapacitors has garnered significant interest in the present decade. The electrode combination offers the benefits of both secondary batteries and electric double-layer capacitors. Various characteristics may influence the performance of hybrid supercapacitors, including the anode pre-lithiation process, anode and cathode materials, electrolyte composition, and electrode design. The performance of hybrid supercapacitors is significantly affected by dendrite formation at the electrode–electrolyte interface, instability of the solid electrolyte interface, and increased thermal detonation concerns [4,5]. Hybrid supercapacitors have issues related to depletion and flammability, often using liquid electrolytes as their electrolyte medium [5,6]. Researchers are now addressing the aforementioned issues by inventing innovative electrolyte materials, including polymer-based and composite electrolytes, as well as inorganic electrolytes that include polymer and ceramic components. A viable alternative to liquid electrolytes, addressing drainage and flammability concerns, is polymer electrolytes [7,8]. Polymer electrolytes, developed from new ion-conducting materials including a polymer host and lithium source, typically exist in a solid or gel state [9]. Despite their limited ionic conductivity, polymer electrolytes are bendable and exhibit superior chemical-thermal stability [10]. Gel polymer electrolytes are receiving significant interest because of their superior conductivity relative to solid-state electrolytes and the absence of the problems associated with organic electrolytes [11]. The creation of a gel polymer electrolyte is a critical process, and researchers have addressed this issue using the homogeneous gelation approach. The polymer host acts as a gelator in the homogeneous gelation process to produce a gel polymer electrolyte when combined with a liquid electrolyte [12,13]. This approach negatively impacts the polymer’s backbone, adversely affecting its electrolyte stiffness properties. A group of researchers used the liquid electrolyte absorption technique. The structure of the polymer is designed as a membrane, enabling the liquid electrolyte to permeate without compromising the mechanical strength of the polymer’s structure. The selection of polymer structure is essential in the fabrication of polymer composite electrolytes, as it enhances the mechanical and ionic conductivity characteristics of the electrolyte. Poly (vinylidene fluoride) (PVDF), poly (ethylene oxide) (PEO), poly (methyl methacrylate) (PMMA), and poly(acrylonitrile) (PAN), together with other polymeric matrices, are used in the fabrication of polymer composite electrolytes. The PVDF polymer is characterized by its remarkable mechanical qualities and superior dielectric constant attributes [14]. PEO has remarkable ionic conductivity [15], while polymer matrices formed from PVDF/PEO blends have improved ionic conductivity properties aimed at 1 mS/cm [16]. PVDF-based active layers are typically fabricated by electrospinning, film/solution casting (including spin coating), and coating methods, each markedly affecting material performance [15]. Electrospinning promotes in situ β-phase formation and high piezoelectric output due to strong electric fields and stretching, whereas casting and spin coating yield uniform dense films but usually require post-stretching or electrical poling to enhance their electroactive properties; coating methods enable scalable, conformal films with performance governed by solvent and thermal processing [17,18]. Researchers have improved the ion conductivity, mechanical stability, and thermal characteristics of PVDF/PEO blends, including plasticized polymer electrolytes, by various methods [19,20,21]. The selection of a suitable electrolyte matrix is paramount for ensuring stable ion transport and mechanical integrity. In this work, the developed PEO/PVDF blend electrolyte demonstrates significant advantages over conventional single-polymer or bio-based systems. PEO provides a highly conductive amorphous medium for efficient ion migration when incorporated with PVDF [19,20]. In 2017, S. Das et al. examined a plasticized polymer electrolyte composed of PVDF-HFP/PEO/EC, achieving an ionic conductivity of around 10^−6^ S/cm [22]. In 2020, Priyanka et al. introduced a plasticized polymer electrolyte composed of PVDF/PEO/LiClO_4_/TiO_2_/PC, demonstrating ionic conductivity within the µS/cm range [23]. In 2023, L. Song et al. synthesized ethylene carbonate plasticized polymer electrolytes. They achieved a peak ionic conductivity of 10^−5^ S/cm [24]. In 2023, N. K. Jaafer et al. developed an ion-conducting polymer electrolyte using CH-g-PMMA in conjunction with ethylene carbonate. The plasticized polymer electrolyte has its greatest ionic conductivity at 1.07 mS/cm [25]. P. Singh et al. produced Plasticized Polymer Electrolytes based on PEMA and ethylene carbonate, focusing on their electrical and structural properties in 2023. The maximum ionic conductivity of the synthesized plasticized electrolyte was 0.46 mS/cm [26]. Nonetheless, the behaviour of conductive ions continues to improve; the ionic conduction mechanism needs to be elucidated. The primary objective of this study is to enhance the ionic conductivity of PVDF/PEO by integrating ethylene carbonate (EC) into a plasticized polymer electrolyte (PPE) and to develop and evaluate a hybrid supercapacitor based on Activated carbon/PPE/Lithium titanate. EC has garnered considerable attention in membrane modification owing to its advantages, including affordability, user-friendliness, environmental adaptability, and seamless membrane formation capabilities [27]. Additionally, the structure of EC contains oxygen atoms with significant electronegative charges. This property enhances the flexibility and amorphous nature of the plasticized polymer electrolyte’s backbone. Moreover, EC was introduced due to its high dielectric constant, which enhances lithium salt dissociation and ionic conductivity, as well as its ability to improve electrode–electrolyte interfacial stability. Furthermore, EC’s film-forming ability imparts the necessary mechanical strength to withstand the physical stresses during battery assembly and cycling without compromising the membrane’s porosity or flexibility. Consequently, using it as a plasticizer can enhance the performance of plasticized polymer electrolytes and hybrid supercapacitors (HSC).

## 2. Materials and Methods

### 2.1. Synthesis of Plasticized Polymer Electrolyte

The subsequent materials were obtained from Sigma Aldrich in Bengaluru, India: (i) poly (ethylene oxide) (PEO), (ii) poly (vinylidene fluoride) (PVDF), (iii) ethylene carbonate (EC), (iv) lithium lanthanum titanate, (v) activated carbon. Glycerol and dimethylformamide (DMF) were supplied by Merck, Mumbai, India. A total of 10 mL of DMF, a polar solvent, and 1 mL of glycerol, a non-solvent, were combined to create a homogeneous polymer solution by combining identical proportions of PEO (50%) and PVDF (50%). Polymer properties are chosen in accordance with the subsequent literature [24]. The EC was combined with the polymer solution in weight percentages of 10%, 20%, 30%, and 40%. The solutions were subjected to heating in a Petri dish at 60 °C for 12 h to yield the solid polymer membrane, which was approximately 200 μM thick. For one hour, the solid polymer composite membranes that were fabricated were submerged in a one-molar solution of LiClO_4_ in an organic electrolyte. Specifically, LiClO_4_ was dissolved at a concentration of 1 M in a mixed solvent system consisting of ethylene carbonate (EC) and diethyl carbonate (DEC) in a volume ratio of [30:70]. The salt was added gradually to the solvent mixture and stirred continuously until a clear and homogeneous solution was obtained prior to gel electrolyte preparation. Excess organic electrolyte was meticulously extracted from the solid polymer composite membrane. Polymer membranes containing 10%, 20%, 30%, and 40% weight percent EC in conjunction with the plasticized polymer electrolyte are denoted by the sample codes PPE10, PPE20, PPE30, and PPE40, respectively. The mass of the moist membranes (M) and dried membranes (M_0_) was determined using the following formula in Equation (1):

(1) $$\delta \left(\%\right)=\frac{M-M_{0}}{M_{0}}\times 100$$

#### 2.1.1. Materials Characterization

An analysis of the functional groups in the plasticized polymer electrolyte was identified within the wavelength of 500 to 4000 cm^−1^ using the Anton Paar, Chile FTIR spectroscopy system. The STG650 TA instruments thermal analyser was used to evaluate the thermal behaviour of the synthesized plasticized polymer electrolyte. The ZEISS upright three-viewer microscope was employed to investigate the structural characterization of the plasticized polymer electrolyte that was synthesized. The adsorption isotherms of N_2_ at 77 K were determined using the fully automated Bruker gas adsorption apparatus. Before analysis, each sample was subjected to out-gassing for two hours at 150 °C under vacuum. The conductivity and dielectric properties of the plasticized polymer electrolyte were derived using silver connecting leads, where ω = 2πf. Here, ω represents the angular frequency, C denotes the capacitance of an empty cell, and tan represents the tangent loss.

(2) σ=l/RA

where A and l are the area and thickness of the membrane, respectively, σ is the ionic conductivity, and R is the resistance obtained from fitting data.

(3) $$\varepsilon^{\prime }=-Z^{\prime }/\omega C(Z^{\prime 2}+Z^{\prime \prime 2})$$

(4) $$\varepsilon^{\prime \prime }=Z^{\prime \prime }/\omega C(Z^{\prime 2}+Z^{\prime \prime 2})$$

(5) $$\tan\delta =\frac{\varepsilon^{\prime \prime }}{\varepsilon^{\prime }}$$

(6) $$\tau =\frac{1}{\omega }$$

During the fabrication of coin cells (2032), activated carbon acts as the cathode, whilst lithium titanate serves as the anode. The electrolyte consists of 10% to 40% of EC containing PPE.

(7) $$C_{s}=\frac{1}{m∆V}\int_{t_{11}}^{t_{2}}I\left(t\right)\times dt$$

(8) $$E_{cell}=\frac{1}{2}C_{sc}{∆V}^{2}\times \frac{1}{4\times 3.6}$$

(9) $$P_{cell}=\frac{E_{cell}}{∆t}$$

Equations (7)–(9) were used for the calculation of charge and discharge curves, where E is the energy density (Wh kg^−1^), C is the measured device capacitance (Fg^−1^), calculated using Equation (9), V is the potential window (V), P is the power density (W kg^−1^), and Δt is the discharge time (s) from the CD curves.

#### 2.1.2. Electrochemical Evaluation

All electrochemical measurements were carried out using an Autolab electrochemical workstation (AutoLab, Chennai, India). The two-electrode coin cell HSC was assembled as a CR2032 coin-type cell with a plasticized polymer electrolyte film (serving as separator and electrolyte). The electrode preparation of the coin cell SC, electrochemical measurement methodologies, and electrochemical calculations are given in the Supplementary Information (SI).

## 3. Results and Discussions

### 3.1. Mechanism of Ethylene Carbonate Plasticized Polymer Electrolyte

**Figure 1:**
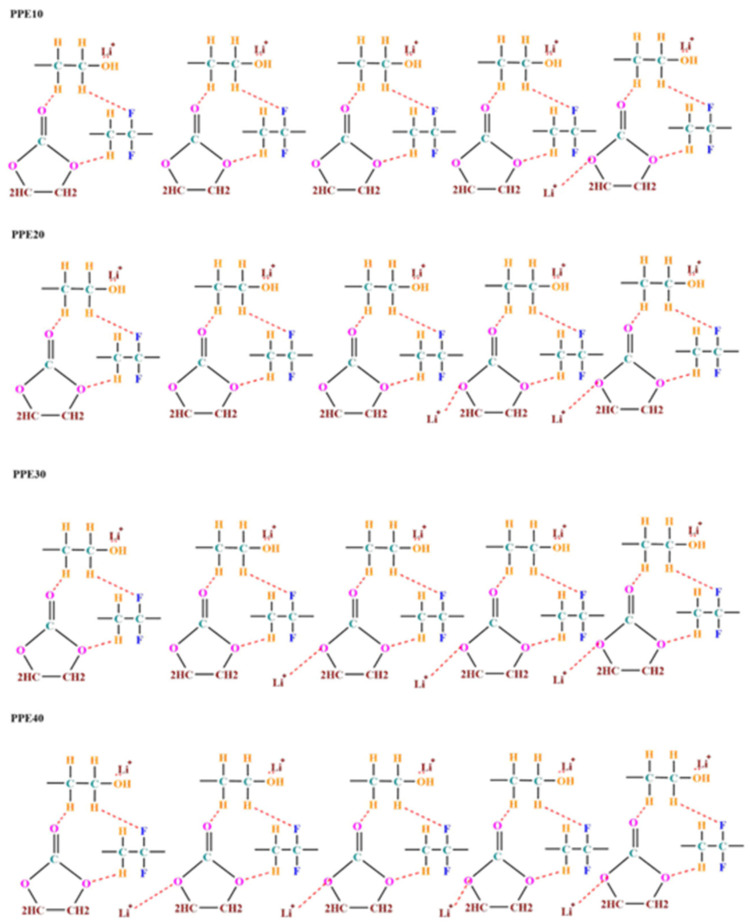
Schematic representation of the ion conduction process in PPE.

Phase inversion technology was used to create a porous polymer composite membrane. The diagram is shown in Figure 1. In polymer blends, PVDF and PEO polymers establish hydrogen bonding interactions. Fluorine’s high electronegativity results in an electrostatic interaction [28]. Furthermore, the EC has three additional oxygen atoms exhibiting pronounced electronegativity characteristics. This tendency influences the polymer backbone via the O-H contact between the polymer and EC, often enhancing its kinetics [29]. The structure of the polymer membrane retains a substantial quantity of liquid electrolyte, resulting in a plasticized polymer electrolyte (PPE). An organic electrolyte dissolved in a DEC/EC solvent containing LiClO_4_ salt dissociates into a Li^+^ cation and a ClO_4_^−^ anion. Li^+^ ions interact with the ether oxygen units of PEO, which possess a high concentration of lone pair electrons, in contrast to the negatively charged oxygen atom of EC. The previously described interactions reduce the route of diffusion for spontaneous Lithium-ion transfer, which tends to increase the conductance of Lithium ions in PPE.

### 3.2. Ftir Spectral Analysis

**Figure 2:**
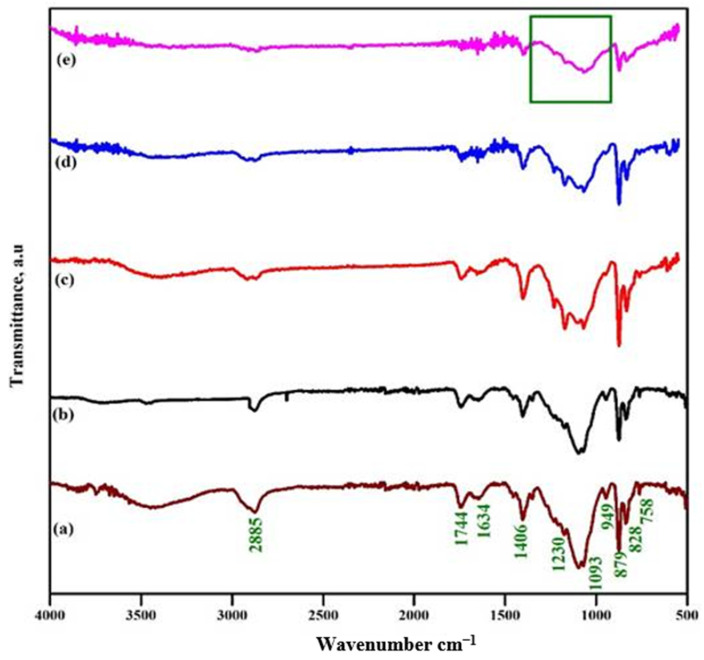
FTIR spectra—PPE: (**a**) EC, (**b**) 10% EC, (**c**) 20% EC, (**d**) 30% EC, and (**e**) 40% EC (The green square box indicates the overlapping of EC vibrations).

The adhesion of EC to polymer composite membranes was investigated using FT-IR spectroscopy to evaluate their molecular vibrations. Figure 2a demonstrates the existence of CH_2_, C=O, -C-, and C-O-C at 2885, 1744, 1634, 1093, and 949 cm^−1^, signifying that the PVDF/PEO consists of polymer mixes in a clear crystalline form [30]. Additionally, the stretching at 1178 and 1406 cm^−1^ is attributed to the C-F and C-H vibrations in polymer blends of PVDF/PEO [31]. The stretching bands at 828 cm^−1^ and 879 cm^−1^ confirm the presence of the β-phase of PVDF in polymer blends of PVDF/PEO [32]. The existence of all bands validates the successful synthesis of polymer blends. The literature indicates that pure ethylene carbonate has stretching at 2998, 2993, 1794, 1765, 1481, 1391, 1152, 1063, 972, 894, 771, and 716 cm^−1^ [33]. The first two vibrations indicate the C-H stretching vibration. The third and fourth bands denote the C=O stretching vibration. The fifth and sixth bands supplant the CH_2_ stretching and bending vibrations. The subsequent three vibration bands represent skeletal stretching, whereas the other two vibration bands correspond to skeletal bending vibrations. The last vibration band signifies the CH_2_ wagging vibrations [34].

Figure 2d illustrates the membrane composed of PPE with a 40-weight proportion of EC. The stretching at frequencies of 2885, 1744, and 1634 cm^−1^ exhibited reduced intensity with increasing EC concentration, indicating that EC influences the polymer backbone via O-H interactions. The hydrogen–fluorine interaction constitutes the bond between ethylene carbonate and polyvinylidene fluoride, since PVDF contains a highly electronegative fluorine atom, while ethylene carbonate includes a hydrogen atom in its composition. The findings are corroborated by the reduced strength of the vibrations at 1406, 828, and 879 cm^−1^. The square symbol signifies a wide spectrum from 1400 to 900 cm^−1^. The polymer mixture and EC vibrational frequencies overlap. An FTIR spectrum verifies that the concentration of EC affected the polymer backbone. This improves the segmental movement and flexibility of PPE [35].

### 3.3. Liquid Electrolyte Uptake Behaviour of PPE

**Figure 3:**
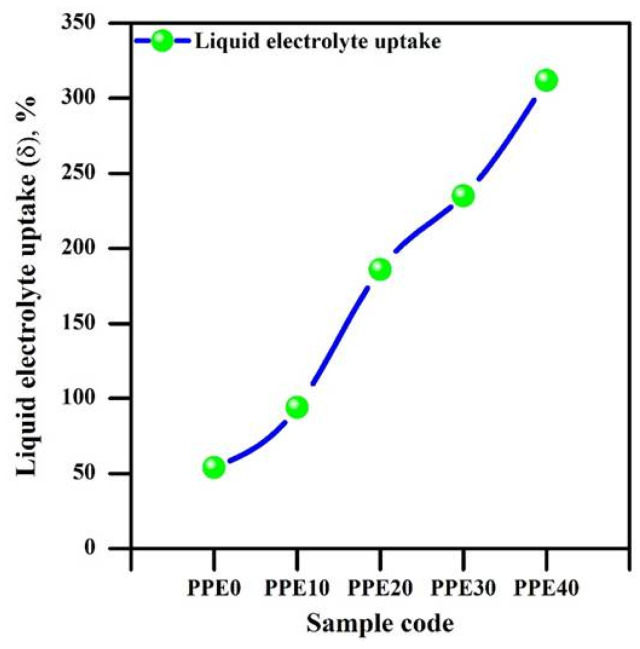
Behaviour of liquid electrolyte absorption of PPE.

Figure 3 illustrates the liquid electrolyte absorption behaviour of polymer composite membranes with varying EC loading from 10 to 40 weight percent. The pore volume and surface morphology of a membrane significantly influence the absorption behaviour of liquid electrolytes. Morphological studies indicate that the incorporation of ethylene carbonate enhances the surface morphology, especially the pore structure, of solid composite material films.

The membrane’s ability to absorb organic electrolyte significantly improved based on the relative weights, leading to enhanced pore capacity. Consequently, the volume of liquid retained by PPE exceeds the quantity of EC in the solid polymer composite membrane [35].

### 3.4. Morphological Studies

**Figure 4:**
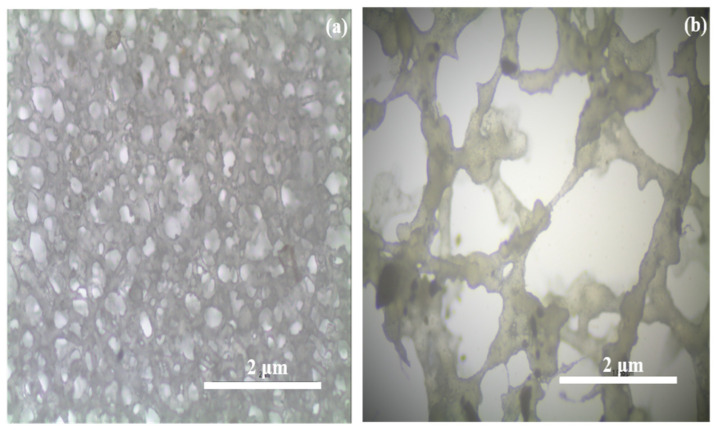
The surface texture of synthesized PPE (**a**) PPE10, (**b**) PPE40.

The surface morphology of PPE10 and PPE40 is shown in Figure 4. The composite PVDF/PEO/EC-based polymer composite membrane’s microscopic picture in Figure 4a demonstrates the porous nature of the membrane. Incorporating more EC weight percent into the polymer blend enhances the pore width. The produced polymer composite membrane can hold more organic electrolytes due to the larger pores in the prepared membrane [36].

### 3.5. Bet Analysis

**Figure 5:**
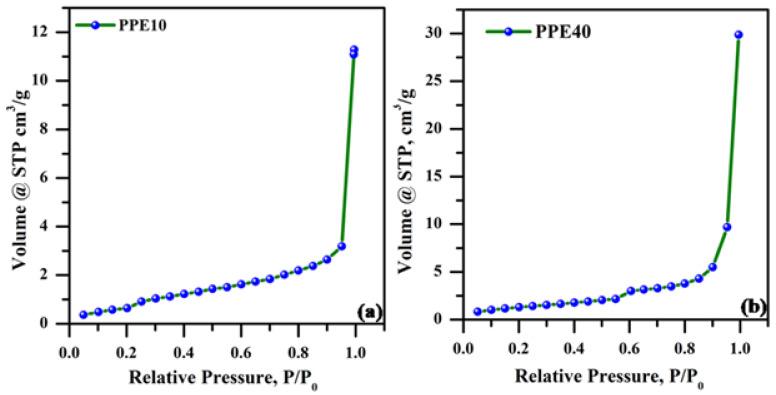
N_2_ adsorption isotherms of plasticized electrolyte (**a**) PPE10 and (**b**) PPE40.

The BET pore studies examined the surface characteristics of plasticized polymer electrolytes. Figure 5a,b illustrates the N_2_ adsorption isothermal characteristics of the plasticized polymer electrolyte. Table 1 presents the surface area, mean pore volume, and pore radius of the plasticized polymer electrolytes PPE10 and PPE40. The BET surface specific area of the PPE40 polymers was determined to be ~3.68 m^2^/g, which was less than that of the PPE 10 polymers (~2.30 m^2^/g). They had a high specific surface area, pore volume, and pore size, as seen in the table. The precise surface area, pore dimensions, and pore volume were improved with the use of a plasticizer. It increases phase separation in polymer electrolytes, hence improving their porous characteristics.

### 3.6. Electrochemical Performance Studies

**Figure 6:**
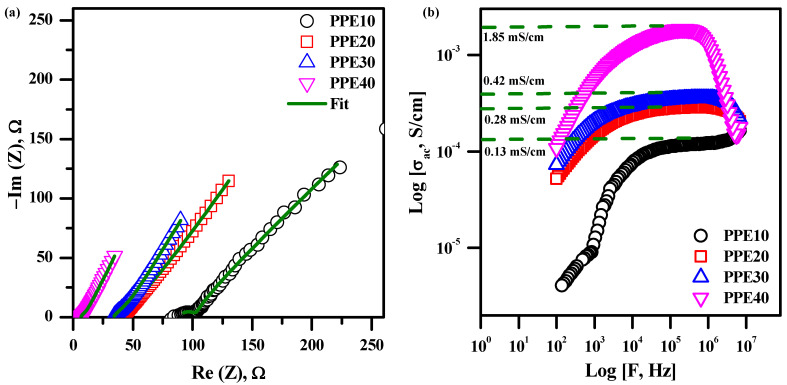
(**a**) Impedance plot of PPE, (**b**) alternating current conductance plot of PPE.

Figure 6a illustrates the PPE impedance curve corresponding to various EC weight ratios at ambient temperature. Electrical parameters, including bulk resistance, capacitance, and phase angle, were extracted from the collected data by complex non-linear least squares fitting. The spectra were fitted using a solution resistance (R_s_) in series with a parallel combination of charge-transfer resistance (R_ct_) and a constant phase element (CPE); a Warburg element was included where necessary to account for ion diffusion at low frequencies. Equation (2) calculates the conductivity values obtained from the fitted parameters, with the results shown in Table 2.

The PPE assesses the performance of an ionic conducting material with relation to a reduced curve at elevated frequency and an inclined plane at subdued frequency [37]. Ion migration, known as the conductivity process that contributes to the resistance of PPE, often aligns with the high-frequency spectrum. The accumulation of ions leads to an increase in the low-frequency state at the electrode/electrolyte contact [38]. The absence of a semicircle in the elevated frequency domain is ascribed to EC, which enhances mobility and ion transport properties. The features of EC enhance charge carrier separation and diminish the resistive qualities of polymer chains. Bulk capacitance is nonexistent in PPE for this reason. Augmenting the concentration of EC in the solid polymer membrane significantly reduces its resistance. The resistance ranges from 100 to 13 Ω. Figure 3 demonstrates that the highly plasticized nature of EC retains a significant quantity of liquid electrolyte while concurrently enhancing the mobility of the polymer chain to augment its flexibility for ionic conduction. The ionic conductivity of 40% EC-based PPE is 2.08 mS/cm, one order of magnitude more than that of 10% EC-based PPE, attributable to the tendency of EC in a membrane to reduce the distribution channel, hence enhancing lithium-ion mobility [39].

### 3.7. Conductivity Studies

**Figure 7:**
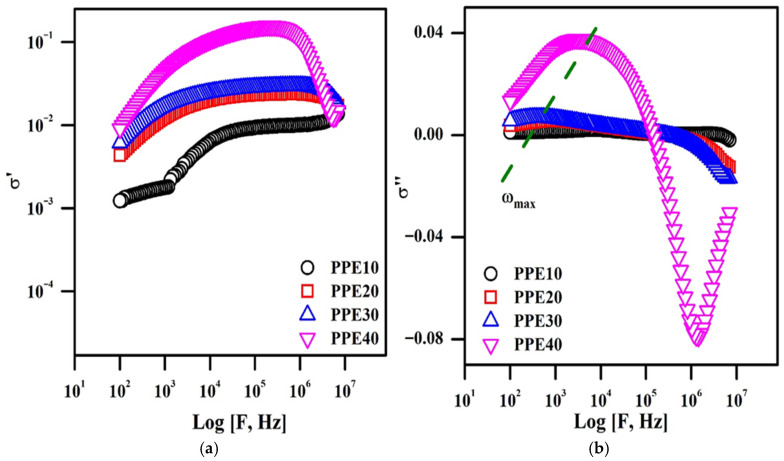
Nonlinear conductance graphs of PPE: (**a**) real conductivity graph, (**b**) imaginary conductivity graph.

Figure 6b depicts a frequency-dependent experiment using PPE and ionic conductivity measurements across various EC weight percentages. The conductivity graph illustrates three separate domains. Region, defined by sub-optimal frequency and conductivity, is the frequency-dependent domain in which ionic conductive behaviour intensifies due to the influence of polarization on frequency. The second state is the frequency-independent flat state, distinguished by ion conductivity resulting from the passage of ions across the material’s extensive conductivity, often including a reduced charge transport time. The ultimate condition is in an elevated-frequency state, characterized by reduced ionic conductivity owing to the polarization of charge carriers, leading to the arrangement of an electric double layer [40]. Moreover, the frequency-independent flat state width expands with the incorporation of EC into the polymer membrane, indicating that the ionic conductivity of the plasticized electrolyte will improve the increased polymer’s flexibility (PPE). Moreover, the AC conductivity measurements align with the conductivity data derived from the impedance graph representation. Figure 7 presents the complex conductivity graphs, demonstrating the correlation between frequency and the real and imaginary components of different concentrations of EC in the polymer membrane. Figure 7a depicts three separate states: The low-frequency state is marked by heightened conductivity due to the arrangement of an electric double layer at the material/electrolyte interface; the frequency-independent state, in which ionic conduction results from the motion of ionic carriers based on their direct current conductivity; and the high-frequency state, known as the dispersion region, where ionic conduction decreases due to the separation of ionic carriers linked to alternating current conductivity. Thus, electrochemical conductivity in polymer electrolytes is generated by short-range ionic transport enabled by the mobility of polymer segments. Figure 7b depicts the imaginary, frequency-dependent aspect of complex conductivity behaviour. The graph illustrated two parameters: the onset frequency (ω_on_) and the maximum frequency (ω_max_). The electrode polarization has its most significant influence at the indicated onset frequency in the picture. Ionic diffusion is governed in a manner analogous to the reduction in conductivity after the onset frequency. The plasticized polymer electrolyte, containing 40% EC, demonstrates improved ω_max_ and ω_on_ values.

### 3.8. Complex Dielectric Analysis

**Figure 8:**
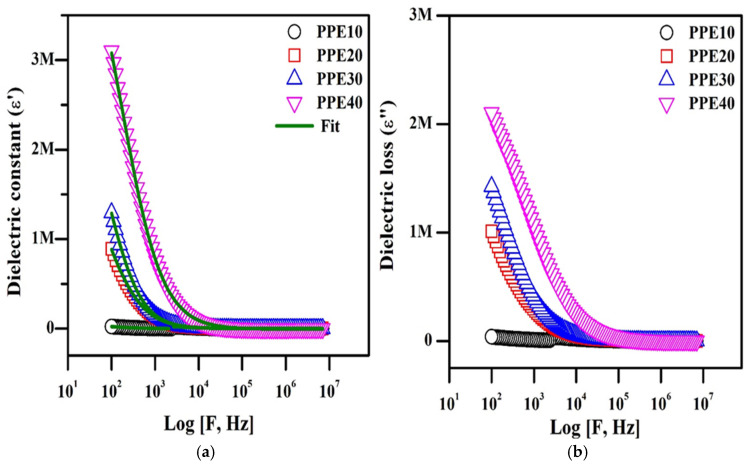
Complex dielectric representations of PPE: (**a**) plot of dielectric constant, (**b**) plot of dielectric loss.

Figure 8a illustrates the dielectric constant of the PPE as influenced by varying weight percentages of EC, whereas Figure 8b depicts the dielectric loss (ε″). The intricate dielectric permittivity is crucial for comprehending phenomena like polarization effects and power related to the dielectric value and loss of dielectrics. Equation (10) was used to get the dielectric constant from the dielectric graph, and the findings are shown in a table.

(10) $$\varepsilon^{\prime }=\varepsilon_{\alpha }+\frac{{(\varepsilon }_{o}-\varepsilon_{\alpha })}{{[1+\left(\omega \tau \right)}^{2(1-\alpha )}]}$$

Table 3 displays the acquired parameters: the constant (ε), loss of dielectric (ε″), the loss of energy, and the lag time. Figure 8a,b demonstrates that increased values in the sub-optimal frequency range of the constant and loss are ascribed to the polarization of charge carriers at the material–electrolyte interfaces and the establishment of a double layer, which augments ionic conductivity. The main reason for this elevated permittivity is the localization of charge carriers. Ion diffusion is affected by dipole orientation relative to the applied field, which is also influenced by reductions in dielectric constant and loss values as frequency rises [41]. The dielectric constant decreases at that frequency due to the alignment of PPE dipoles with the electric field. The inclusion of EC in the polymer composite membrane often increases the dielectric constant by augmenting the quantity of ions and their mobility and diffusivity, according to the properties of the charges. The difference between the inactive and dynamic dielectric constants is used to determine its strength [42]. The concentration of EC in the polymer composite determines the relaxation strength of PPE in relation to ionic mobility and relaxation duration. The adaptability of polymer chains and their localized motion enhances mobility. The unobstructed lithium-ion pathway with little relaxation time is often abridged to improve the electrical conductivity of the plasticized electrolyte.

### 3.9. Derivative Loss (Δ)

**Figure 9:**
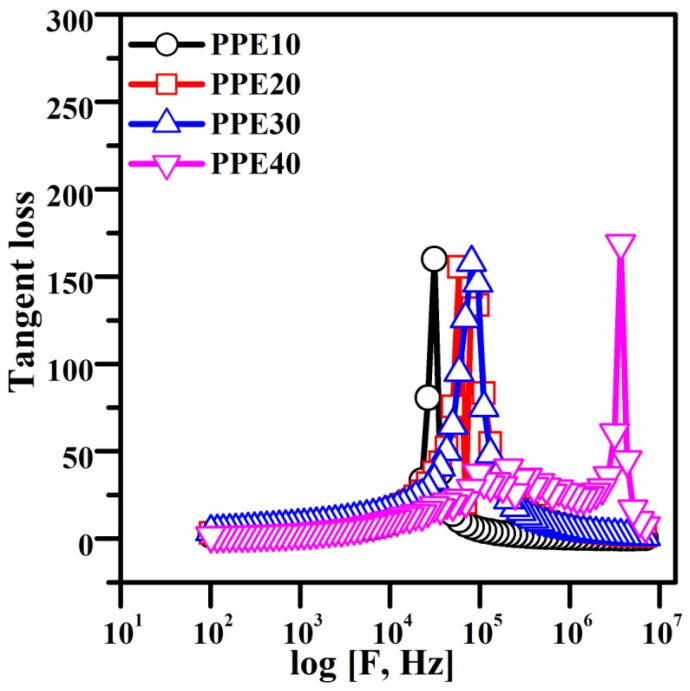
Plot showing tangent loss for PPE.

Figure 9 illustrates the Tangent loss associated with different weight percentages of EC in the PPE. This results from the separation of charge carriers and ionic movement, directly measuring the energy released.

An increased buildup of lithium ions occurs at the contact due to the frequency of the applied field. Minimizing the relaxation period expedites lithium-ion diffusion, thus improving conductivity and dielectric constant in the high-frequency spectrum. The maximum intensification at 40 wt.% EC in the plasticized polymer electrolyte indicates the synergistic formation of PPE by decreasing the electrode resistance. The plasticized properties of EC facilitate a decrease in resistivity by improving the flexibility of the electrolyte. This enhances the plasticity of the electrolyte structure and increases the dynamics of the metameric electrolyte [43]. This behaviour reduced the relaxation time of the PPE, as seen in Table 2.

### 3.10. Electric Modulus of Complexity

**Figure 10:**
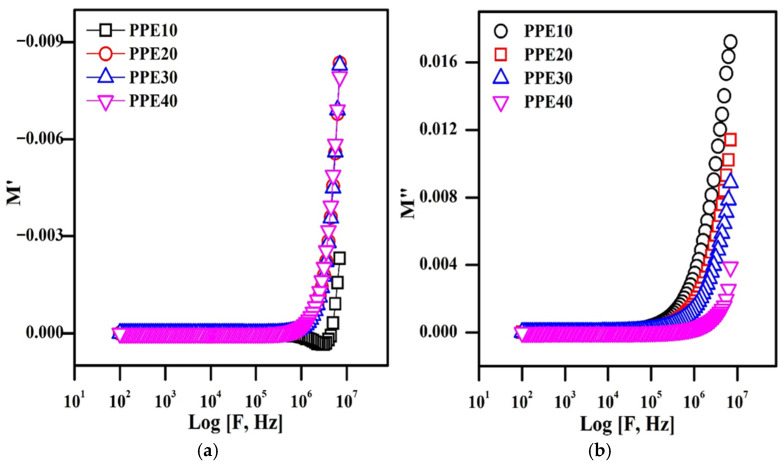
Complex electric modulus representations of PPE: (**a**) real electric modulus graph, (**b**) imaginary electric modulus graph.

To study the ion kinetics and conductivity relaxation behaviour in plasticized electrolytes, an electric complexity analysis is used, which is a crucial spectrum. The reciprocal of the complex dielectric permittivity is the complex electric modulus, which is denoted by the formula M = 1/ε*. At various EC weight ratios, Figure 10 displays the frequency-dependent real component M′ and imaginary part M″ of the plasticized polymer electrolyte. As a result, M′ and M″ have zero values at low frequencies and behave nonlinearly at high frequencies [44,45].

The non-Debye relaxation phenomena may be characterized by asymmetric modulus behaviour at high frequencies. Furthermore, because of the increase in charge carrier mobility and diffusivity, the values of M″ exhibit a change in plasticity in the elevated frequency region. The plasticization of the polymer composite membrane by 40% EC diminishes the polymer’s resistive properties, thus enhancing ionic mobility and decreasing relaxation duration, which often results in improved energy loss.

### 3.11. Thermal Analysis

**Figure 11:**
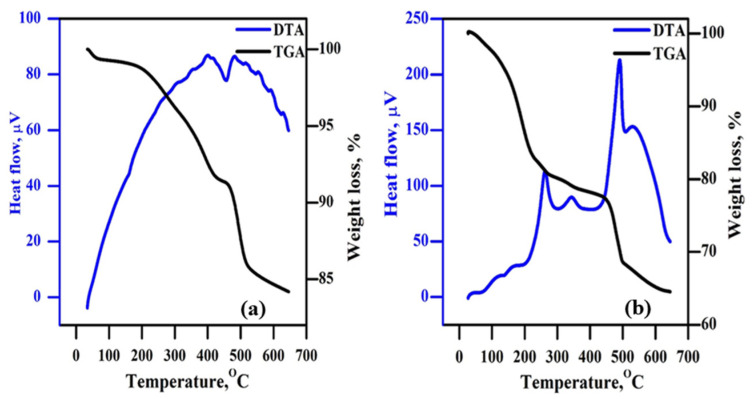
(a) Examination of PPE using thermogravimetric and differential thermal methods (**a**) for PPE10 and (**b**) for PPE40.

Figure 11 illustrates the results of analyzing the TGA and DTA curves of the plasticised PPE10 and PPE40 polymer electrolytes, which reveal their thermal characteristics. Both samples show minimum weight loss due to the loss of water molecules up to 140 °C. At 200 °C, PPE10 starts to degrade thermally, whereas PPE40 begins to degrade at 95 °C. This is a result of the EC failing. Because the polymer composites in the PPE10 sample had less EC substitution, the degradation of the PEO started at 208 °C. In addition, it is clear that the PVDF started to degrade in both samples at around 450 °C [28].

### 3.12. Dc Polarization Method

**Figure 12:**
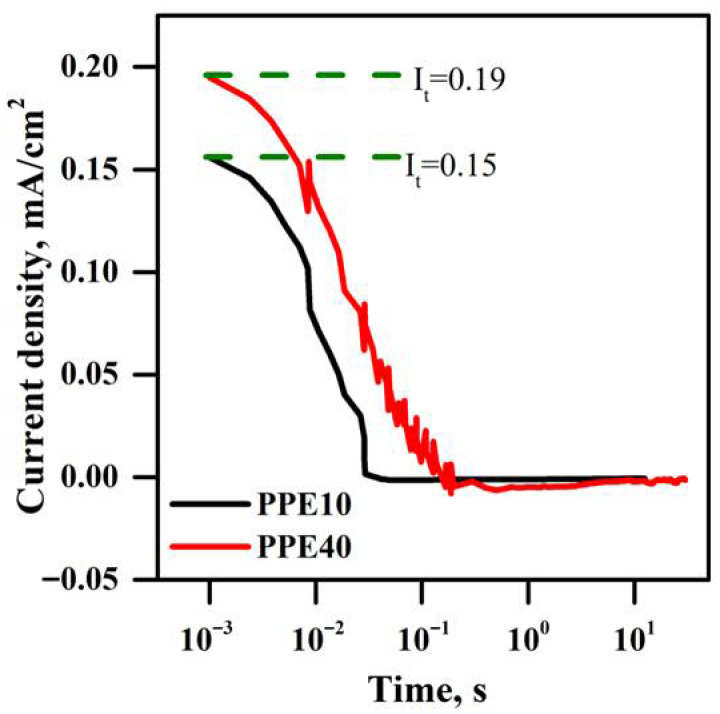
Ion transfer numbers of synthesized PPE: PPE10 (black colour line), and PPE40 (red colour line).

Figure 12 depicts the ion transfer number (tin) of the PPE, as determined by DC polarization techniques. PPEs are positioned between two blocking electrodes, namely silver electrodes, and subjected to the application of 0.5 V during this characterization (DC polarization voltage). In PPE, the ions are displaced with the application of voltage. A delayed temporal response is caused by the blocking electrode’s constraint on ion mobility and the exclusive passage of electrons. The resulting current includes the movement of both charge carriers, ions and electrons. The electric current, which is minimal in PPE, is generated by the motion of electrons. The PPE10 and PPE40 samples both have an ion transfer number of 0.99. In comparison to the PPE10 sample, the PPE40 sample exhibits the highest total current. Conductivity tests have shown that ethylene carbonate enhances ionic conductivity, which explains this behaviour.

### 3.13. Fluctuation of Ionic Mobility, Number Density, and Mobility with PCEs

Ionic diffusion, movement, and overall density were ascertained by dielectric properties and AC conductivity spectra; this connection is used to calculate diffusivity. The Einstein equation specifies the number density, with D representing diffusivity, σ indicating ionic conductivity, K denoting Boltzmann’s constant, T signifying absolute temperature, and q as the elementary charge constant. A correlation between numerical density and electrical conductivity may be used to ascertain mobility [46]. Table 2 presents a compilation of the calculated values. The composite membrane, which comprises 40% EC, incorporates a plasticised polymer electrolyte, demonstrating a low charge carrier concentration with increased ionic mobility and diffusivity [47,48]. The EC attracts Li-ions owing to its electronegativity and diminishes their concentration. Nonetheless, their ionic mobility and diffusivity are enhanced owing to their plasticized characteristics [49,50].

### 3.14. Characteristic of Fabricated Hybrid Supercapacitors (HSC)

**Figure 13:**
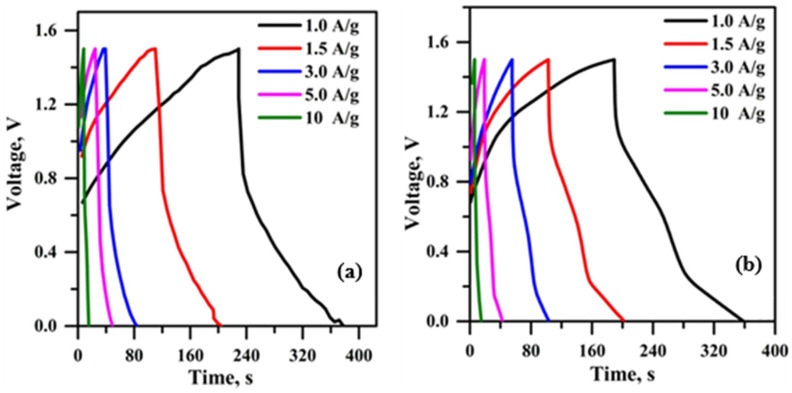
GCD of PPE-based (**a**) PPE10 and (**b**) PPE40.

Figure 13 illustrates the plotted GCD curves of hybrid supercapacitors using micro-porous solid-state polymer electrolyte at varying current densities. The charge curves of the constructed hybrid supercapacitor exhibit asymmetry relative to the matching discharge curves, indicating a considerable degree of reversibility in these devices [4,46]. Micro-porous solid-state polymer electrolytes have a promptly accessible interface, uniformly distributed framework, and increased active sites that facilitate the acceleration of redox processes during the charge–discharge cycle [50]. Figure 13a illustrates the GCD curves of the constructed hybrid supercapacitor using PPE10. This hybrid supercapacitor has a reduced discharge time (180 s) at a current density of 1 A/g compared to a hybrid supercapacitor using PPE, attributable to the higher ionic conductivity of the PPE40 electrolyte (2.08 mS/cm). The specific capacitance value of hybrid supercapacitors is shown in Table 4.

The data indicate that both hybrid supercapacitors exhibit a decline in specific capacitance as current density increases, attributed to inadequate ion diffusion and sub-optimal electrode–electrolyte interaction. However, the PPE40-containing hybrid supercapacitor exhibits a lesser decline in specific capacitance compared to the PPE10-containing hybrid supercapacitor. This outcome is ascribed to the effective ion diffusion and the establishment of the electrode–electrolyte interface in PPE40. The maximum specific capacitance of 110 F/g at a current density of 1 A/g is attained by the PPE40-based hybrid supercapacitor, attributed to the superior ion conduction properties of PPE40. The determined specific capacitance values exceed the previously documented value (Table 5). To evaluate the relationship between energy and power density, a Ragone plot was generated (Figure S1) for the PPE-based hybrid supercapacitors. Because of the high specific capacitance, the PPE40-based hybrid supercapacitor achieved a maximum energy density of 35 Wh kg^−1^ at a power density of 752 W kg^−1^. In comparison, the PPE10-based hybrid supercapacitor exhibited a slightly lower energy density of 30 Wh kg^−1^ at a power density of 745 W kg^−1^. These results demonstrate that the PPE40 configuration optimizes the trade-off between energy storage capacity and rapid power delivery.

**Figure 14:**
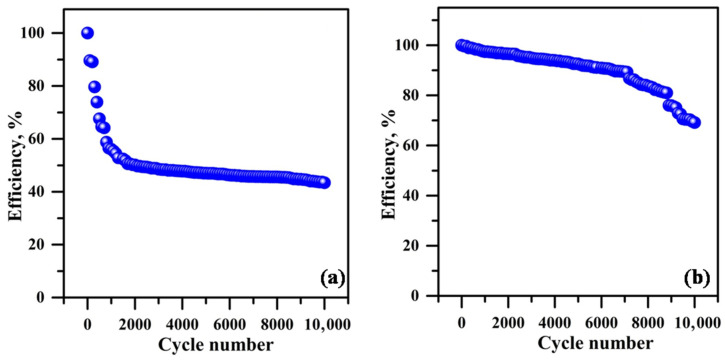
Cycle number vs. cycle efficiency plot (**a**) PPE10-based hybrid supercapacitor and (**b**) PPE40-based hybrid supercapacitor.

**Figure 15:**
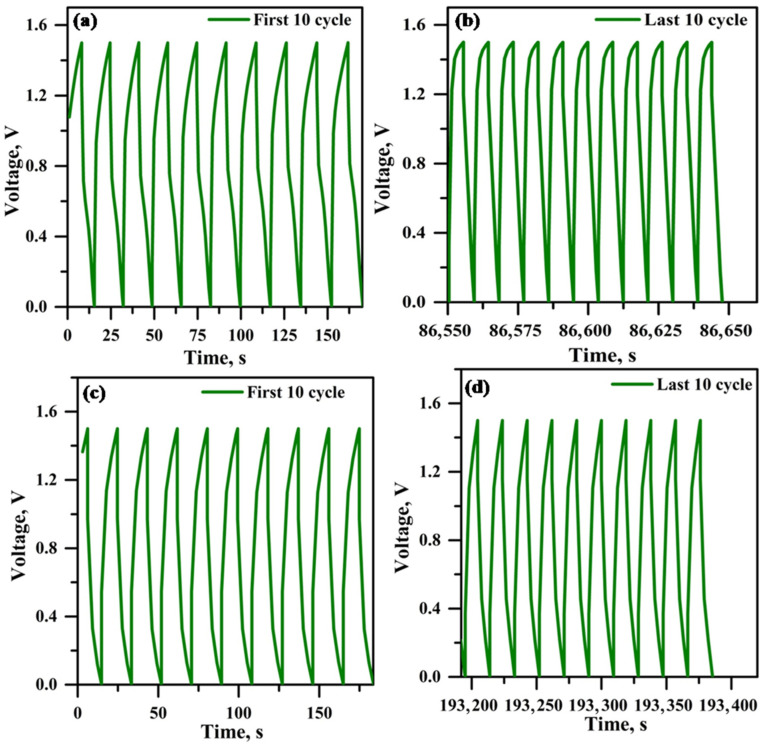
Initial and last 10 cycles of chronopotentiometry (**a**,**b**) EC/PPE10 (**c**,**d**) EC/PPE40-supercapacitor.

Figure 14 illustrates the efficiency plot (for every 100 cycles) against the cycle number. Figure 14a illustrates the hybrid supercapacitor using PPE10, whereas Figure 14b depicts the hybrid supercapacitor employing PPE40. The efficiency decline seen after 10,000 cycles in the hybrid supercapacitor using PPE10 is attributed to inadequate interfacial contact at the electrode and electrolyte interface. The hybrid supercapacitor using PPE40 exhibits reduced efficiency deterioration owing to little interfacial resistance at the electrode–electrolyte contact throughout the cycle, as corroborated by impedance spectroscopy findings. Figure 15a,b and Figure 15c,d illustrate the initial and final ten-cycle GCD curves of the hybrid supercapacitor using PPE10 and PPE40, respectively. Both hybrid supercapacitors exhibit elevated discharge currents during the first 10 cycles and decreased discharge currents in the last 10 cycles. These data correspond closely to the efficiency vs. cycle number graph.

**Figure 16:**
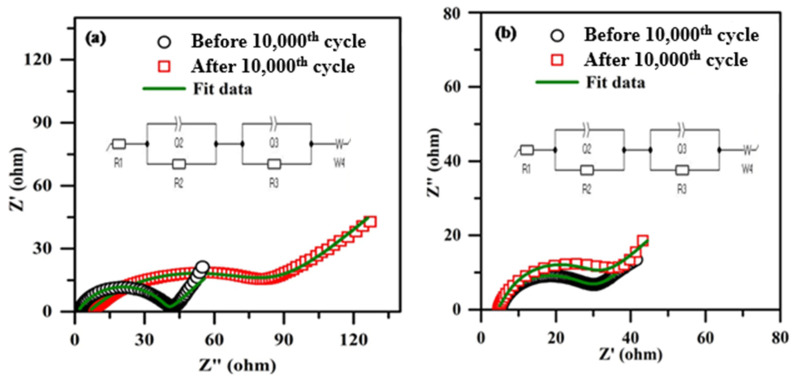
Electrochemical Impedance spectroscopy of (**a**) EC/PPE10, (**b**) EC/PPE40.

Figure 16 depicts the impedance spectra of hybrid supercapacitors using micro-porous solid-state polymer electrolytes, before and after cycling. A comparable electrical circuit is proposed in the inset of the figure. The suggested equivalent circuit derives the interfacial resistance value from the experimental data. In an analogous circuit, R1 represents the shunt resistance, also referred to as the connecting wire resistance; R2 denotes the electrolyte or bulk resistance; and R3 signifies the electrode/electrolyte interface resistance, also known as interfacial resistance. Owing to the superficial characteristics of the electrolyte and electrode, Q1 and Q2 are the phase constant components. W4 refers to the Warburg resistance resulting from ion diffusion. Both hybrid supercapacitors exhibit elevated R3 values after 10,000 cycles. This augmentation is attributed to the connection of advancement between electrolytes and electrodes in all composite supercapacitors [51,52,53].

Figure 16b indicates that the hybrid supercapacitor using PPE40 exhibits the lowest R3 value in comparison to the hybrid supercapacitor employing PPE10, attributable to the elevated concentration of EC, which improves the uniformity of the plasticized polymer electrolyte and facilitates optimal electrode/electrolyte interface development [54,55].

## 4. Conclusions

The phase inversion method effectively fabricated a micro-porous solid-state polymer electrolyte using PVDF/PEO/EC. A plasticized polymer electrolyte was formed due to the significant amount of liquid electrolyte encapsulated inside the porous framework of PVDF/PEO/EC (PPE). The inclusion of EC significantly increased the conductivity of ions and dielectric behaviour of the plasticized polymer electrolyte, diminishing the resistive attributes of polymer chains, and the plasticized nature of EC generally increased the capacity for motion and diffusion of charge carriers within the plasticized polymer electrolyte. Consequently, at ambient temperature, PPE40 has an exceptional ionic conductivity of 2.08 mS/cm. A hybrid supercapacitor using PPE40 exhibited superior specific capacitance (112 F/g), power density (752 W/kg), and energy density (35 Wh/kg) compared to a hybrid supercapacitor based on PPE10.

## Figures and Tables

**Table 1:** Surface area, average pore volume, and pore radius of PPE.

S. No.	Sample Code	Specific Surface Area (m^2^/g)	Pore Size (nm)	Pore Volume (cm^3^/g)
1	PPE10	2.30	14.3	1.64
2	PPE40	3.68	34.3	4.62

**Table 2:** Conductivity parameters of prepared plasticized PPE.

S. No.	Sample Code	σ_dc_ (S/cm)	τ (s)	D (cm^−2^s^−1^)	µ (cm^−2^V^−1^s^−1^)	*n* (cm^−3^)
1.	PPE10	0.28 × 10^−3^	1.05 × 10^−6^	2.91 × 10^−6^	1.18 × 10^−4^	6.54 × 10^19^
2.	PPE20	1.20 × 10^−3^	5.34 × 10^−7^	2.68 × 10^−6^	1.08 × 10^−4^	8.90 × 10^18^
3.	PPE30	1.75 × 10^−3^	4.77 × 10^−7^	3.04 × 10^−6^	1.23 × 10^−4^	6.15 × 10^18^
4.	PPE40	2.08 × 10^−3^	5.04 × 10^−8^	5.23 × 10^−5^	2.11 × 10^−3^	5.82 × 10^18^

**Table 3:** Dielectric parameters of prepared plasticized PPE.

S. No.	Sample Code	ε_α_	ε_o_	Δε	τ (µs)	A
1.	PPE10	1731	0.17 × 10^6^	0.18 × 10^6^	0.090	0.86
2.	PPE20	1552	2.09 × 10^6^	2.09 × 10^6^	0.010	1.03
3.	PPE30	955	2.78 × 10^6^	2.78 × 10^6^	0.011	1.16
4.	PPE40	7887	4.75 × 10^6^	4.75 × 10^6^	0.005	0.96

**Table 4:** Specific capacitance of plasticized PPE-based hybrid supercapacitors.

S. No.	Device Name	Electrochemical Parameters	1000 mA/g	1500 mA/g	3000 mA/g	5000 mA/g	10,000 mA/g
1	PPE10	Specific Capacitance (F/g)	95	78	62	51	39
		Specific Power density W/kg	745	1125	2250	3825	7564
		Specific Energy density (Wh/kg)	30	24	19	16	12
2	PPE40	Specific Capacitance (F/g)	112	102	96	80	66
		Specific Power density W/kg	752	1125	2250	3750	7425
		Specific Energy density (Wh/kg)	35	32	30	25	20

**Table 5:** Evaluation of the specific capacitance values with existing literature for polymer electrolytes.

S. No.	Polymer System	Current Density (A/g)	Specific Capacitance (F/g)	Ref.
1	Celgard 2400 Micro-porous membrane, North California USA) with 1 M Li_2_SO_4_ electrolyte	0.2	83	[46]
2	A leaf-vein-like MnO_2_@PVDF nanofiber gel polymer electrolyte	0.5	20	[47]
3	Porous poly (acrylonitrile-polyhedral oligomeric silsesquioxane) membrane	2.0	88	[48]
4	Celgard 2400 Micro-porous membrane	0.1	70	[49]
5	PVDF/PEO/10 wt.% of EC-based plasticized polymer electrolyte	1.0	95	Present work
6	PVDF/PEO/40 wt.% of EC-based plasticized polymer electrolyte	1.0	112	Present work

## Data Availability

The original contributions presented in this study are included in the article/Supplementary Materials. Further inquiries can be directed to the corresponding authors.

## Supplementary Materials

The following supporting information can be downloaded at: https://www.mdpi.com/article/10.3390/polym18040485/s1. The Fabrication of coin cell explained in SI. The Ragone Plot comparison of PPE10 & PPE40 based hybrid super-capacitors in Figure S1.
