# Supplementary material for: Combined Effects of PVDF/PEO-EC GEL Polymer Electrolytes for High-Performance Hybrid Electrochemical Supercapacitors
Source: Polymers (Basel). 2026 Feb 14;18(4):485. doi: 10.3390/polym18040485 (PMC12943987; doi:10.3390/polym18040485)
Supplement: Supplementary file 1 [file polymers-18-00485-s001.zip › polymers-4097219-supplementary.pdf]

Electronic supplementary information

Combined Effects of PVDF/PEO-EC GEL Polymer Electrolytes for High-Performance Hybrid Electrochemical Supercapacitors

*Ramkumar Gurusamy[a], Tae Hwan Oh[b], Arunpandian Muthuraj[b] and *Aravindha Raja Selvaraj c**

[a]Department of Renewable Energy Science, ManonmaniamSundaranar University, Tirunelveli 627 012, Tamil Nadu, India.

[b]School of Chemical Engineering, Yeungnam University, Gyeongsan 38541, Republic of Korea.

[c]School of Chemical, Biological and Battery Engineering, Gachon University, Seongnam-Si 13120, Republic of Korea.

*Corresponding author(s): rajanano12@gmail.com; muthurajarunpandian1523@yu.ac.kr

Fabrication of Coin cell Hybrid supercapacitor (HSC):

The hybrid supercapacitor was assembled in a CR2032 coin-cell configuration inside an argon-filled glove box. The electrodes were formed from the thick slurry and then the active material coated on the electrodes were cut into discs of 16 mm diameter and used as positive and negative electrodes with average mass of 2mg. The assembly sequence consisted of the activated carbon electrode (anode), plasticized polymer electrolyte film (serving as separator and electrolyte), and Lithium titanate oxide LTO electrode (cathode), followed by stainless steel spacers and a spring. The electrochemical performance and ionic dynamics were investigated via Electrochemical Impedance Spectroscopy (EIS). The measurements were conducted over a frequency range spanning from 1 Hz to 7 MHz to capture both the bulk electrolyte resistance and the interfacial charge-transfer processes.

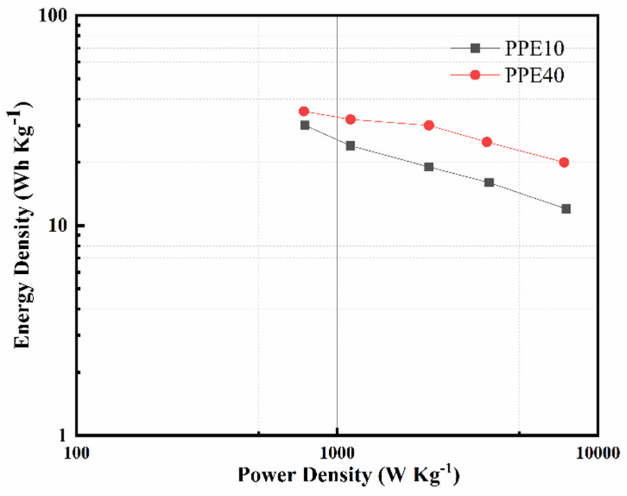

Figure. S1: Ragone Plot comparison of PPE10 & PPE40 based hybrid super-capacitors.
